# TLR signaling adaptor protein MyD88 in primary sensory neurons contributes to persistent inflammatory and neuropathic pain and neuroinflammation

**DOI:** 10.1038/srep28188

**Published:** 2016-06-17

**Authors:** Xing-Jun Liu, Tong Liu, Gang Chen, Bing Wang, Xiao-Lu Yu, Cui Yin, Ru-Rong Ji

**Affiliations:** 1 Jiangsu Province Key Laboratory of Anesthesiology, School of Anesthesiology, Xuzhou Medical University, Xuzhou, Jiangsu, 221004, China; 2 Department of Anesthesiology, Duke University Medical Center, Durham, North Carolina, 27710 USA; 3 Jiangsu Key Laboratory of Translational Research and Therapy for Neuro-Psycho-Diseases, Institute of Neuroscience, Soochow University, Suzhou, Jiangsu, 215021, China; 4 Department of Neurobiology, Duke University Medical Center, Durham, North Carolina, 27710 USA

## Abstract

Increasing evidence suggests that neuro-immune and neuro-glial interactions are critically involved in chronic pain sensitization. It is well studied how immune/glial mediators sensitize pain, but how sensory neurons control neuroinflammation remains unclear. We employed *Myd88* conditional knockout (CKO) mice, in which *Myd88* was deleted in sodium channel subunit Na_v_1.8-expressing primary sensory neurons, to examine the unique role of neuronal MyD88 in regulating acute and chronic pain, and possible underlying mechanisms. We found that baseline pain and the formalin induced acute inflammatory pain were intact in CKO mice. However, the late phase inflammatory pain following complete Freund’s adjuvant injection and the late phase neuropathic pain following chronic constriction injury (CCI), were reduced in CKO mice. CCI induced up-regulation of MyD88 and chemokine C-C motif ligand 2 expression in DRG neurons and macrophage infiltration into DRGs, and microglia activation in spinal dorsal horns in wild-type mice, but all these changes were compromised in CKO mice. Finally, the pain hypersensitivity induced by intraplantar IL-1β was reduced in CKO mice. Our findings suggest that MyD88 in primary sensory neurons plays an active role in regulating IL-1β signaling and neuroinflammation in the peripheral and the central nervous systems, and contributes to the maintenance of persistent pain.

Accumulating evidence suggests that neuro-immune and neuro-glial interactions play a critical role in chronic pain sensitization^1^,^2^. Toll-like receptors (TLRs) are type I transmembrane proteins that detect components of foreign pathogens (PAMP, pathogen-associated molecular pattern) and endogenous ligands (DAMP, danger-associated molecular pattern) released by stressed cells that are key players in neuro-immune and neuro-glial interactions in neurological and neuropsychiatric conditions including chronic pain^3^,^4^,^5^,^6^. TLRs are expressed in a variety of immune cells including mast cells, B cells, regulatory T cells, macrophages and dendritic cells that regulate innate and adaptive immunity^7^. The myeloid differentiation factor-88 adaptor protein (MyD88) mediates most TLRs (except for TLR3) signaling, as well as Toll/Interleukin receptor domain signaling through the interleukin (IL)-1 and IL-18 receptors^7^. TLRs regulate innate immunity via activation of the NF-κB and MAPK pathways and subsequent production of inflammatory cytokines and chemokines^3^,^7^. Functional TLRs are also expressed in microglia and astrocytes that modulate glial activation in persistent pain and itch conditions^8^,^9^,^10^. While it is well known how immune/glial mediators sensitize pain, little is known as to how sensory neurons control inflammation and neuroinflammation^11^.

Of great interest TLRs such as TLR3, TLR4, TLR5, and TLR7 are also expressed in primary sensory neurons in dorsal root ganglia (DRGs) of the peripheral nervous system that regulates sensory function, such as pain or itch^10^,^12^,^13^,^14^,^15^,^16^. MyD88 is also expressed in nociceptive DRG neurons and has been implicated in chemotherapy-induced neuropathic pain in rats^8^. In addition to nociceptive neurons, Na_v_1.8 is also expressed in low-threshold A-fibers (e.g., Aβ fiber) DRG neurons^17^,^18^. However, research on sensory neuron-specific role of TLR signaling is hampered by the lack of tissue (e.g., sensory neuron) specific knockout mice. To this end, we generated *MyD88* conditional knockout (CKO) mice by deleting *MyD88* selectively in sodium channel Na_v_1.8-expressing nociceptive neurons^19^. We found that paclitaxel induced innate and adaptive immunity in DRGs was impaired in CKO mice^19^. In this study, we further investigated the unique role of neuronal MyD88 in acute vs. chronic pain. Here we show that selective deletion of *Myd88* in Na_v_1.8-expressing primary sensory neurons in CKO mice leads to reductions in complete Freund’s adjuvant (CFA) induced inflammatory and chronic constriction injury (CCI) induced neuropathic pain in the maintenance phase, without affecting basal pain and acute inflammatory pain. Further, we show that MyD88 is required for CCI induced up-regulation of the chemokine C-C motif ligand 2 (CCL2, also named as monocyte chemoattractant protein-1 (MCP-1)) in DRG neurons. Finally, macrophage infiltration in DRGs and microglia activation in spinal dorsal horns after CCI are reduced in CKO mice.

## Results

### Primary deletion of Myd88 adaptor protein in small-sized DRG neurons in CKO mice

**Figure 1:**
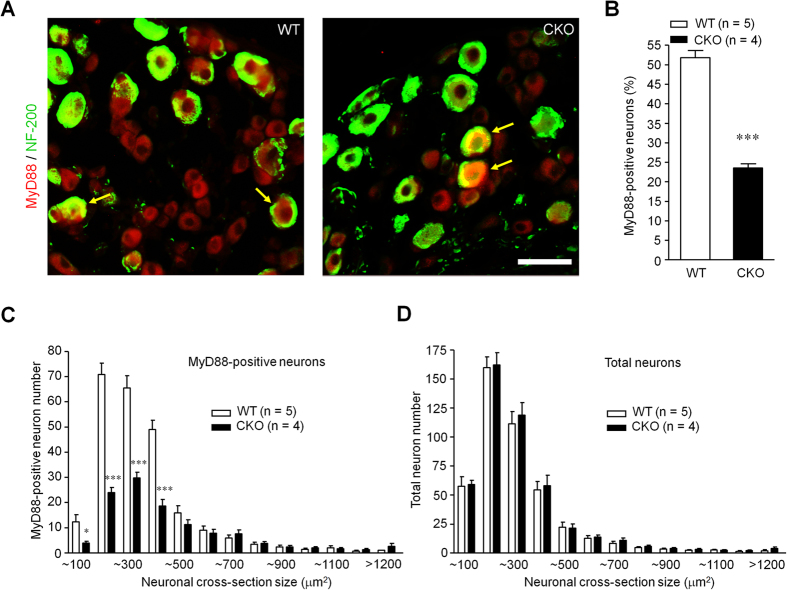
Deletion of Myd88 adapter protein in small-sized DRG neurons of CKO mice. (**A**) Double immunostaining showing MyD88 localization in DRG neurons of littermate control (WT) and MyD88 conditional knockout (CKO) mice. Arrows indicate neurons with co-localization of MyD88 and NF200. Scale, 50 μm. (**B**) Percentage of MyD88-positve neurons in WT and MyD88 CKO mice. Six DRG sections were included per mouse. ***P < 0.001, two-tailed unpaired student T-test compared with WT; n = 4~5 mice/group. **(C,D)** Size distribution of MyD88-positive neurons (**C**) and total neurons (**D**) in WT and MyD88 CKO mice. Six DRG sections were counted per mouse. *P < 0.05, ***P < 0.001, two-way ANOVA compared with WT; n = 4~5 mice/group.

We first examined the expression of MyD88 in DRG neurons of littermate mice (WT) and MyD88 CKO mice, in which Myd88 protein was deleted in Na_v_1.8-expressing primary sensory neurons, as described in our previous study^19^. Double immunostaining showed that MyD88 was expressed in majority DRG neurons, including both small-sized C-fiber nociceptive neurons (NF-200^−^) and some large-sized A-fiber neurons (NF-200^+^, Fig. 1A) in WT mice. While the MyD88 expression was unaltered in large diameter (NF-200^+^) DRG neurons, its expression was dramatically reduced in small-sized (NF-200^−^) neurons in CKO mice (Fig. 1A,C). The quantitative analysis from immunohistochemistry showed that the percentage of MyD88^+^ neurons was reduced from ~52% (2368/4550) in WT DRGs to ~24% (1373/5559) in CKO DRGs (two-tailed unpaired T-test, t = 13.44, *P* < 0.0001) (Fig. 1B). Furthermore, size frequency analysis revealed that MyD88 immunoreactivity was mainly localized in small-sized DRG neurons (Fig. 1C). Size frequency analysis also showed loss of MyD88 is small DRG neurons (two-way ANOVA, F_(1,249)_ = 74.48, *P* < 0.0001, Fig. 1C). By contrast, the size distribution of total DRG neurons did not change in CKO mice, suggesting that there is not developmental defect of DRG neurons in CKO mice (Fig. 1D). Although our data showed MyD88 loss in small-sized DRG neurons in naïve CKO mice, MyD88 loss may also occur in some large Aβ fiber neurons under inflammatory pain condition. Indeed, functional upregulation of Na_v_1.8 was found in Aβ fiber neurons after inflammation^17^.

### Baseline pain and acute inflammatory pain are normal in CKO mice

**Figure 2:**
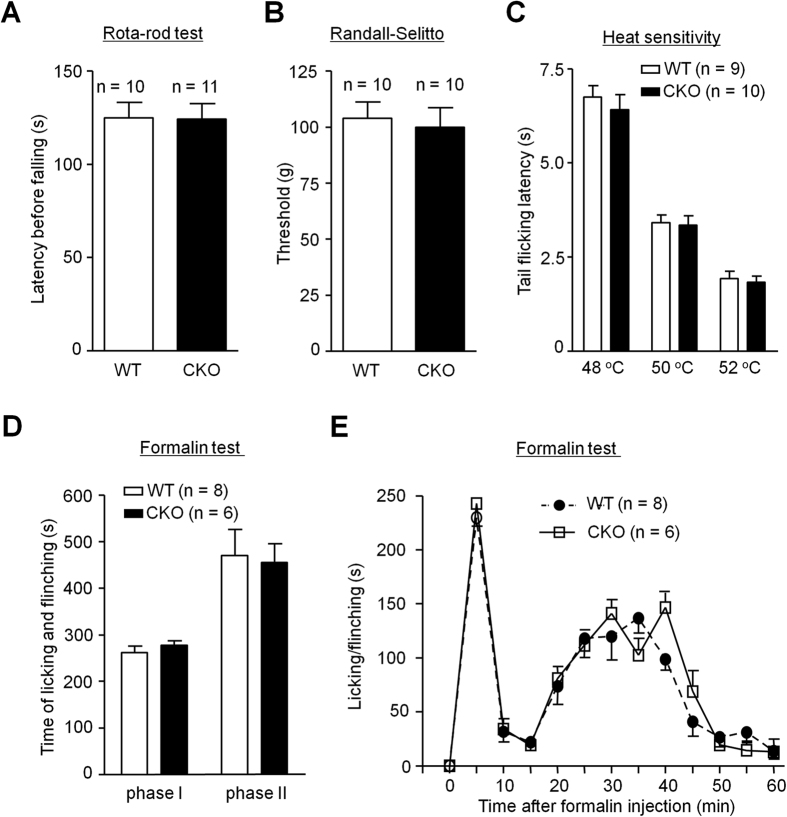
Sensory neuron MyD88 is not required for motor function, baseline pain and acute inflammatory pain. **(A–C**) Baseline pain was intact in CKO mice. (**A**) Motor function, revealed by falling latency in Rotarod test. (**B**) Mechanical pain sensitivity, assessed by tail withdrawal threshold in Randall-Selitto test. (**C**) Heat pain in tail flick test. n = 9~11 mice/group. **(D,E)** Formalin-induced acute inflammatory pain is intact in WT and MyD88 CKO mice. (**D**) The total flinch and licking time and (**E**) the time course of 5% formalin-induced pain behavior. n = 6~8 mice/group.

We subsequently examined whether depletion of MyD88 in nociceptive neurons would affect normal pain perception (baseline pain sensitivity) and acute inflammatory pain. CKO mice displayed normal motor function in Rota-rod test (Fig. 2A). CKO mice also have intact baseline pain, by exhibiting normal mechanical and thermal nociception in the Randall-Selitto test and tail-flick test (Fig. 2B,C), respectively. We also tested formalin induced acute inflammatory pain in WT and CKO mice. Both the first phase (0–10 min) and the second phase (10–60 min) nocifensive responses were unaltered in CKO mice (Fig. 2D,E). Two-way ANOVA of Phase II response revealed no significant effect of phenotype (F_(1,108)_ = 0.27, *P* = 0.616, Fig. 2E). These results suggest that MyD88 in primary sensory neurons is not necessary for generating acute pain.

### Persistent inflammatory pain and neuropathic pain are reduced in CKO mice

**Figure 3:**
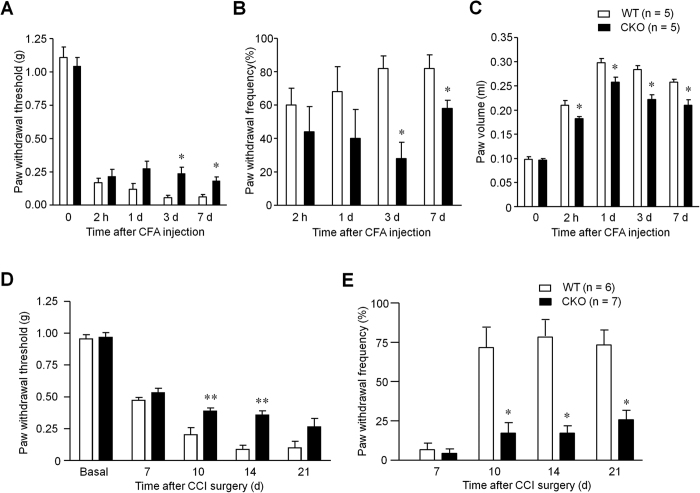
Sensory neuron MyD88 contributes to CFA induced persistent inflammatory pain and CCI induced neuropathic pain in the late phase. (**A,B**) Complete Freund’s adjuvant (CFA) induced persistent inflammatory pain, expressed as mechanical hyperalgesia (**A**) and allodynia (**B**, percentage of response to 0.16 g filament) in WT and MyD88 CKO mice. *P < 0.05, two-way ANOVA compared with WT; n = 5 mice/group. (**C**) CFA induced paw edema as indicated by paw volume. (**D,E**) Nerve injury (CCI) induced mechanical hyperalgesia (**D**) and allodynia (**E**), expressed as 50% paw withdrawal threshold (**D**) and frequency response to a von Frey filament stimulation (0.16 g, **E**) in WT and MyD88 CKO mice. *P < 0.05, **P < 0.01, two-way ANOVA compared with WT, n = 5~7 mice/group.

**Figure 4:**
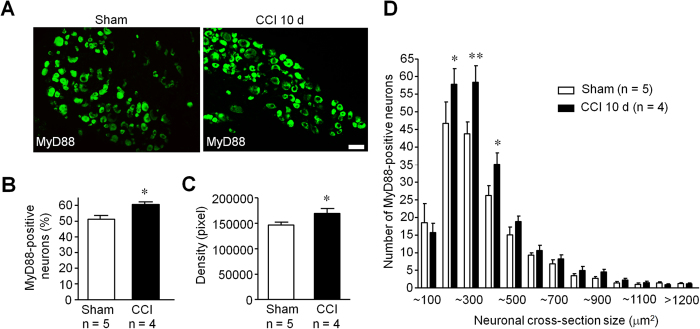
Immunohistochemistry shows up-regulation of MyD88 in DRG neurons of CCI mice. (**A**) Immunohistochemistry showing the expression of MyD88 in DRG neurons of sham and CCI mice 10 d after surgery. Scale, 50 μm. (**B,C**) Quantification analysis from immunohistochemistry results. (**B**) percentage of MyD88-positive neurons; (**C**) density of MyD88-positive neurons. Six DRG sections were counted per mouse. *P < 0.05, two-tailed unpaired student t-test compared with sham control; n = 4~5 mice/group. (**D**) Size distribution of MyD88-positive neurons in CCI and Sham surgery control mice (10 d). Six DRG sections were analyzed per mouse. *P < 0.05, **P < 0.01, two-way ANOVA compared with sham control; n = 4~5 mice/group.

Next, we examined persistent inflammatory pain induced by intraplantar injection of CFA and persistent neuropathic pain induced by CCI in WT and CKO mice. Notably, CFA induced mechanical allodynia was only impaired in the maintenance phase (3 d and 7 d) but not in the induction phase (2 h and 1 d) in CKO mice (Fig. 3A,B). Two-way ANOVA revealed significant effect of phenotype (F_(1,32)_ = 8.81, *P* = 0.018, Fig. 3A; F_(1,24)_ = 5.9, *P* = 0.041, Fig. 3B). CFA induced paw edema was also reduced in CKO mice (two-way ANOVA, F_(1,32)_ = 36.49, *P* = 0.0003, Fig. 3C). Furthermore, CCI induced mechanical allodynia in the maintenance phase (10, 14 and 21 d) but not in the induction phase (7 d) was reduced in CKO mice (Fig. 3D,E). Two-way ANOVA revealed significant effect of phenotype (F_(1,55)_ = 19.84, *P* = 0.001, Fig. 3D; F_(1,44)_ = 34.16, *P* = 0.001, Fig. 3E). Immunohistochemistry assay also confirmed the increased expression of MyD88 in DRG neurons 10 d after CCI (Fig. 4A), assessed by both percentage of positive neurons (50.1% (2465/4863): 60.1% (2480/4125)), two-tailed unpaired t-test, t = 3.16, *P* = 0.004, Fig. 4B) and intensity of immunostaining (two-tailed unpaired t-test, t = 2.26, *P* = 0.034, Fig. 4C). Size frequency analysis revealed that increased MyD88 immunoreactivity was mainly localized in small-sized DRG neurons 10 d after CCI (two-way ANOVA, F_(1,275)_ = 8.22, *P* = 0.0045, Fig. 4D). Together, these results suggest that selective deletion of *Myd88* in DRG neurons contributes to the maintenance of chronic inflammatory and neuropathic pain.

### MyD88 in primary sensory neurons contributes to cytokine IL-1β evoked pain in mice

**Figure 5:**
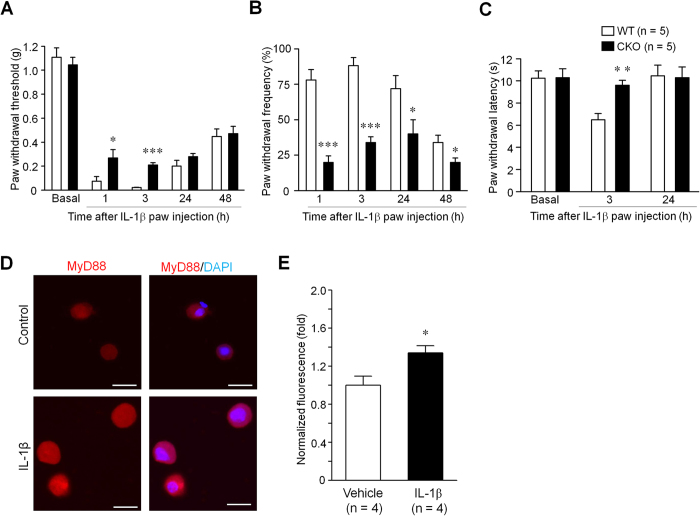
Sensory neuron MyD88 contributes to intraplantar IL-1β induced pain hypersensitivity. **(A–C**) Intraplantar injection of IL-1β induces sub-acute inflammatory pain, expressed as mechanical hyperalgesia (expresses as 50% paw withdrawal threshold; **A**), allodynia (expresses as frequency response to 0.16 von Frey hair stimulation; **B**) and heat hyperalgesia (**C**) in WT and MyD88 CKO mice. *P < 0.05, **P < 0.01, ***P < 0.001, two-way ANOVA compared with WT, n = 5 mice/group. **(D**,**E)** IL-1β increases MyD88 expression in cultured DRG neurons. **(D)** Immunocytochemistry of MyD88 in cultured primary DRG neurons after exposure to IL-1β (10 ng/ml) or vehicle for 3 h. Scale, 20 μm. **(E)** Normalized immunofluorescence of MyD88-positive neurons in DRG culture. *P < 0.05, two-tailed unpaired T-test, compared with vehicle control; n = 4 culture slides.

As MyD88 was demonstrated to mediate downstream IL-1 receptor signaling^20^, we next investigated IL-1β induced pain behaviors in WT and CKO mice. Intraplantar injection of IL-1β was sufficient to evoke enhanced pain states, including mechanical allodynia and heat hyperalgesia (Fig. 5A–C). Interestingly, IL-1β induced mechanical allodynia was significantly reduced in CKO mice (two-way ANOVA, F_(1,32)_ = 14.28, *P* = 0.007, Fig. 5A; F_(1,24)_ = 55.48, *P* < 0.001, Fig. 5B). IL-1β induced heat hyperalgesia was also significantly reduced in CKO mice (two-way ANOVA, F_(1,16)_ = 7.96, *P* = 0.004, Fig. 5C). These results suggest an active role of neuronal MyD88 in regulating IL-1β signaling in persist pain.

To test the mechanisms by which MyD88 regulates IL-1β signaling within DRG neurons, we cultured DRG neurons and exposed them to IL-1β (10 ng/ml) for 3 h. As shown in Fig. 5D, MyD88 immunoreactivity was increased in DRG neurons after IL-1β treatment, compared with vehicle control. Two-tailed unpaired T-test of statistical analysis revealed a significant effect of phenotype (t = 2.775, *P* = 0.0391, Fig. 5E). This result suggests that IL-1β may enhance pain via MyD88 expression in DRG neurons.

### MyD88 in primary sensory neurons contributes to neuronal CCL2 expression and macrophage activation/infiltration in DRGs

**Figure 6:**
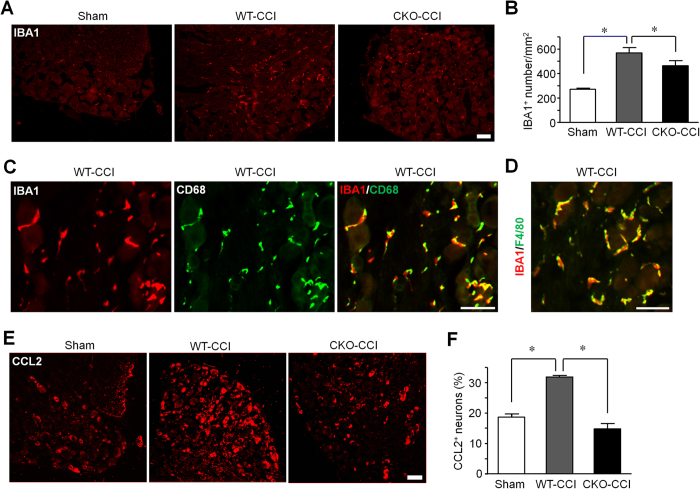
Sensory neuron MyD88 is necessary for the CCI induced CCL2 expression in DRG neurons and IBA1 expression in DRG macrophages. (**A**) Immunostaining of IBA1 showing macrophages/monocytes in DRGs of WT mice following sham surgery and CCI (10 d) in WT and MyD88 CKO mice. Scale, 100 μm. (**B**) Quantification of IBA1^+^ macrophages in DRGs of (**A**) as number per square mm. (**C,D**) Double immunostaining of IBA1/CD68 (**C**) and IBA1/F4/80 (**D**) in DRGs of CCI mice (10 d). Scale, 50 μm. (**E**) CCL2 immunostaining in DRGs of WT mice following sham surgery and CCI and in CKO mice after CCI (10 d). Scale, 100 μm. (**F**) Percentage of CCL2-positive DRG neurons shown in (**E**). Scale, 50 μm. Three DRG sections were analyzed per mouse. *P < 0.05, one-way ANOVA, compared with sham or WT; n = 4~6 mice/group.

Chemokines such as CCL2 have been implicated in neuroinflammation and chronic pain sensitization^21^. CCL2 was shown to increase Na_v_1.8 activity and excitability of DRG neurons^21^,^22^. Notably, CCL2 expression was found to be dependent on MyD88 in murine mammary carcinomas cells^23^. We postulated that primary sensory neurons recruit macrophages to DRGs through chemokine release. Nerve injury not only induced the activation and infiltration of IBA1-expressing macrophages in DRGs but also caused the up-regulation of CCL2 in DRG neurons (Fig. 6A,B,E,F). Of interest CCI induced IBA1 expression in DRGs was compromised in CKO mice (one-way ANOVA, F_(2,12)_ = 18.75, *P* = 0.0002, Fig. 6B). Further characterization of macrophages showed that IBA1 is largely co-localized with macrophage markers CD68 or F4/80 in DRGs of CCI mice (Fig. 6C,D). Finally, CCI induced CCL2 expression in DRG neurons, which was compromised in CKO mice (One-way ANOVA, F_(2,6)_ = 53.17, *P* = 0.0002, Fig. 6E,F). These results suggest that MyD88 in primary sensory neurons contributes to neuronal CCL2 expression and macrophage activation/infiltration in DRGs of chronic neuropathic pain mice.

### MyD88 in primary sensory afferents contributes to microglia activation in spinal dorsal horn

**Figure 7:**
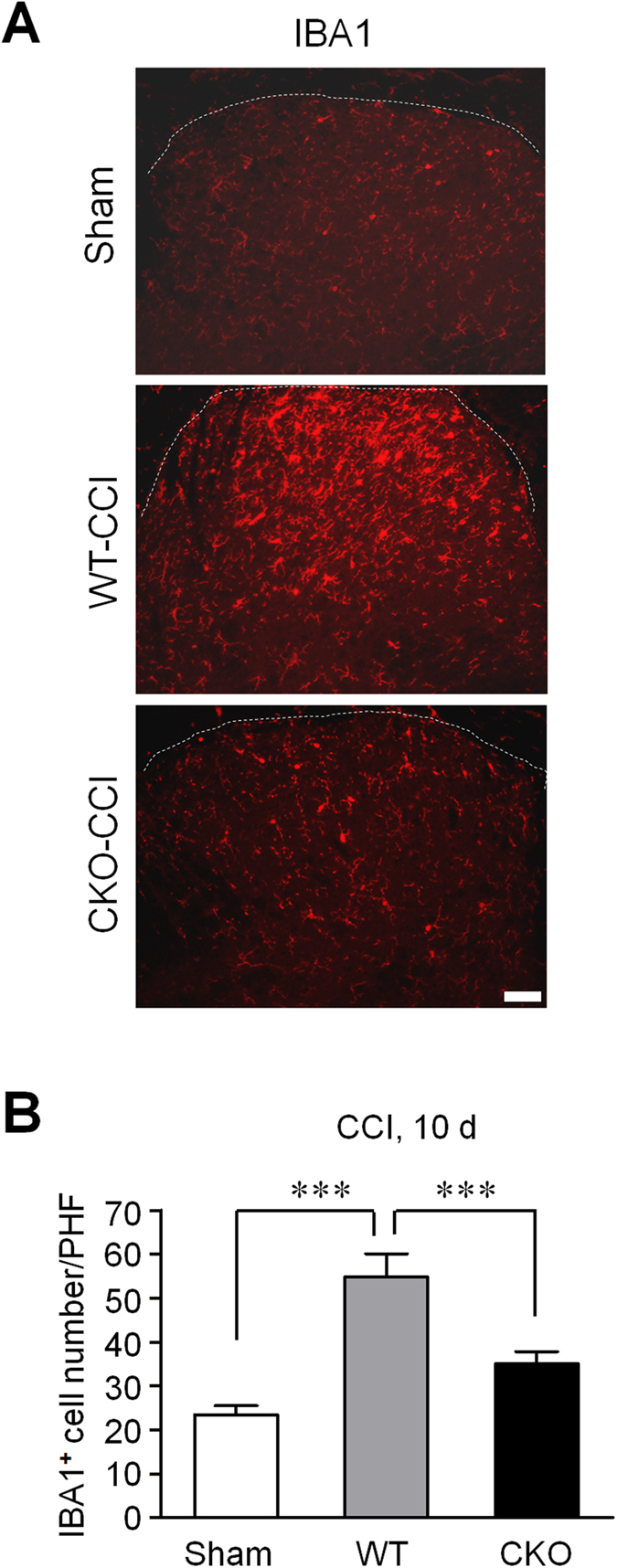
MyD88 in primary sensory afferents contributes to microglia activation in spinal dorsal horns after nerve injury. (**A**) Immunostaining of IBA1 showing microglia activation in spinal dorsal horns of WT mice after sham and CCI surgery (10 d) and MyD88 CKO mice after CCI surgery (10 d). Scale, 100 μm. (**B**) Quantification of IBA1^+^ microglia in spinal dorsal horns of (**A**) as number of per high field (PHF). Three spinal dorsal horn sections were analyzed per mouse. ***P < 0.001, one-way ANOVA compared with sham; n = 4~5 mice/group.

Since primary sensory neurons project to the spinal dorsal horn, we test whether MyD88 in primary sensory afferents regulates the microglia activation in spinal dorsal horn. As expected, we found more IBA1-positive microglia in the spinal dorsal horns of WT mice post CCI procedure compared with sham mice. However, there are less activated microglia in CKO mice compared with WT mice (Fig. 7A,B). One-way ANOVA of statistical analysis revealed a significant effect of phenotype (F_(2,8)_ = 45.54, *P* = 0.0213, Fig. 7B). This result suggests that MyD88 contributes to microglia activation in spinal cords of mice post CCI procedure.

## Discussion

Increasing evidence suggests that neurons expecially primary sensory neurons express TLRs, including TLR3, TLR4, TLR5, TLR7^10^,^12^,^13^,^14^,^15^,^16^. TLR3, TLR4, and TLR7 are mainly expressed by small diameter C-fiber neurons that are nociceptive or/and pruriceptive^10^,^12^,^14^, whereas TLR5 is primarily expressed in large diameter Aβ neurons that are essential for eliciting mechanical allodynia in neuropathic pain^13^. MyD88 is primarily expressed in small diameter DRG neurons^8^, although some large-sized neurons also express MyD88 (Fig. 1A,C). Intrathecal injection of MyD88 inhibitor inhibited chemotherapy induced neuropathic pain^8^. Consistently, spinal nerve ligation induced neuropathic pain was reduced in *Myd88* KO mice^24^. Although these studies support an important role of MyD88 in neuropathic pain, they are not able to prove a specific role of nociceptor MyD88 in acute and chronic pain. Also, global *Myd88* deficiency results in both cognitive and motor impairments in mice^25^. Therefore, we generated *Myd88* CKO mice in which *Myd88* was deleted in Na_v_1.8^+^ sensory neurons (Fig. 1A)^19^. Our data showed that motor function, basal pain perception, and formalin induced acute inflammatory pain were all intact in CKO mice. Consistently, MyD88 has limited involvement in TLR7 mediated TRPA1 activation and acute pain following miRNA let-7b stimulation^26^.

Another interesting finding of this study is that late phase but not early phase inflammatory and neuropathic pain was reduced in CKO mice. This result is consistent with that of global deletion of *Myd88*^24^. Furthermore, TLR7 ligand let-7b induced persistent mechanical allodynia but not acute pain depends on MyD88^26^. It is conceivable that MyD88 in DRG neurons controls late phase inflammatory and neuropathic pain via gene regulation, which may explain the delayed effects in inflammatory and neuropathic pain in MyD88 CKO mice (Fig. 3A–E). In particular, MyD88 is required for nerve injury induced upregulation of CCL2, in DRG neurons (Fig. 6E,F), consistent with another report in which the release of CCL2 was dependent on MyD88 pathway in murine mammary carcinomas cells^23^. Previous studies showed that CCL2, upregulated in DRG neurons after nerve injury^27^, could increase Na_v_1.8 activity and excitability of DRG neurons^22^, leading to an enhanced neuropathic pain state. In addition, CCL2 could enhance neuropathic pain via neuron-macrophage interaction in DRGs. We postulate that secretion of CCL2 from DRG neurons could cause activation of resident macrophages and infiltration of circulating macrophages to DRG tissue. Interestingly, CCI induced IBA1 expression, a marker for macrophage, which co-expresses with CD68 or F4/80 (Fig. 6C,D), was also abrogated in CKO mice (Fig. 6A,B). In another study, global deletion of MyD88 reduced IBA1-positive macrophage infiltration by 50% in dorsal horn of spinal cord following L5 spinal nerve ligation^21^,^24^. Thus, MyD88 mediated CCL2 expression in primary sensory neurons also regulates neuroinflammation in DRGs (Fig. 6A,B) and in spinal cord (Fig. 7). However, the contribution of sensory neuron-MyD88 to overall inflammation could be limited, since we only found moderate reduction in CFA induced edema in CKO mice (Fig. 3C). Given the marked deficits in innate and adaptive immunity in the peripheral nervous system^19^, sensory neuron-MyD88 may maintain chronic pain, especially neuropathic pain, by controlling neuroinflammation in DRGs and spinal cord.

We also found that pain hypersensitivity induced by intraplantar IL-1β was reduced in CKO mice (Fig. 5A–C), suggesting that neuronal MyD88, presumably expressed by nerve terminals in hindpaw skin, contributed to IL-1β produced pain. Indeed, IL-1 receptor is also expressed in DRG neurons, and IL-1β was shown to rapidly and directly activate nociceptors to generate action potentials^28^. Since intraplantar IL-1β induced mechanical allodynia and heat hyperalgesia was reduced in the early phase (1 and 3 h) in CKO mice, MyD88 in nerve terminals may regulate IL-1β induced acute pain via posttranslational modification that occurs within minutes or tens of minutes. Interestingly, recent research has shown different immune cells mediate pain hypersensitivity in male and female mice, and male dependent TLR4 signaling in the spinal cord^29^. Although male mice were used in the present study, female animals are worth testing in the future.

In summary, our findings have demonstrated that MyD88 expressed by Na_v_1.8^+^ primary sensory neurons contributes to persistent inflammatory pain and neuropathic pain in the maintenance phase, by regulating CCL2 expression in DRG neurons and neuroinflammation in the peripheral and central nervous systems (e.g., macrophage activation and infiltration in DRGs, and microglia activation in spinal cord). MyD88 in sensory neurons is also required for intraplantar IL-1β induced acute pain via possible posttranslational regulation. Together, our data further support an important role of the TLRs/MyD88 pathway in sensory neuron that detects and responds to both exogenous pathogens (PAMP) and endogenous danger signals (DAMP) in chronic pain.

## Materials and Methods

### Animals

MyD88^flox^ mice were purchased from Jackson Labs. Na_v_1.8^cre^ transgenic mice were kindly provided by Rohini Kuner (University of Heidelberg)^30^. Breeding colonies were maintained by mating MyD88^f/f^ with Na_v_1.8^cre^-MyD88^f/f^ mice as previous reported^19^. The homozygous conditional knockout mice (MyD88^f/f^ with Na_v_1.8^cre^) referred to as CKO mice, whereas the MyD88^f/f^ littermates were used as wild type control mice (WT). All animal experiments were conducted in accordance with the National Institutes of Health Guide for the Care and Use of Laboratory Animals. All the experimental procedures with animals were approved by the Institutional Animal Care & Use Committee (IACUC) of Duke University. Adult male mice (>8~10 weeks) were used and kept under a 12-hour light/dark cycle. Animals were habituated to the testing environment daily for at least two days before behavior experiments. WT and CKO mice were not randomized. All the behavioral tests and tissue quantification were done by individuals who were blinded to the treatment or genotypes of the mice. Young male mice (4~6 weeks) were used for DRG neuron culture.

### Reagents

We purchased CFA, formalin, IL-1β, paraformaldehyde, DNAse I and Opti MEM culture medium from Sigma-Aldrich Company (St. Louis., MO). Collagenase, trypsin inhibitor and dispase-II were provided by Roche Diagnostics (Mannheim, Germany). B27 supplement was purchased from Invitrogen Company (Carlsbad, CA).

### Behavioral tests

#### Motor function test

A Rota-rod system (IITC Life Science Inc., Woodland Hills, CA) was used to assess the animal motor function. Mice were tested for three trails separated by 10 min intervals. During the tests, the speed of rotation was accelerated from 2 to 20 r.p.m. within 3 min. The falling latency was recorded and averaged.

#### Randall-Selitto test

We used Randall-Selitto Analgesy-meter (Ugo Basile, Comerio-Varese, Italy) to examine mechanical sensitivity by applying ascending pressure to the tail of a mouse and determined the mechanical pain threshold when animal showed a clear sign of discomfort or escape, with a cut-off threshold of 250 g to avoid tissue damage.

#### Tail immersion test

Tail immersion test was used to assess heat pain sensitivity by keeping the tail of a mouse in hot water at 48, 50, or 52 °C and recorded the tail flick latency, with a cut-off time of 10 seconds.

#### Acute inflammatory pain model

Mice were given intraplantar injection of 10 μl of diluted formalin (5% in saline) to induce acute inflammatory pain. The spontaneous pain behavior was video-recorded and the duration of licking and flinching was measured in a blinded manner in 5 min bins for 60 min after the formalin injection.

#### Persistent inflammatory pain model

Mice were given intraplantar injection of 20 μl CFA to induce persistent inflammatory pain.

#### Paw edema measurement

To assess the CFA induced inflammation and edema, paw volume was determined by water displacement plethysmometer (Ugo Basile).

#### Neuropathic pain model

To induce neuropathic pain, we performed CCI model^31^. Under isoflurane anesthesia, the sciatic nerve of mouse was exposed and then three ligatures (6–0 prolene) were ligated around the nerve proximal to the trifurcation. The ligatures were loosely tied and a distance between each ligature was one millimeter. The sham group received the same surgery but without nerve ligation.

#### Intraplantar injection of IL-1β induced pain

Mice were given intraplantar injection of 10 μl IL-1β (10 ng) for behavioral test. Mechanical allodynia and thermal hyperalgesia were tested by using von Frey test and Hargreaves test, respectively.

#### Von Frey test

Mice were put in a box on elevated metal mesh floor and stimulated hind-paw with a series of von Frey hairs with logarithmically incrementing stiffness (0.08–2.00 grams, Stoelting, Wood Dale, IL). The hairs were presented perpendicular to the plantar surface, and determined the 50% paw withdrawal threshold (PWT) using Dixon’s up-down method. For testing mechanical allodynia, we also checked paw withdrawal frequency in response to a subthreshold von Frey hair stimulation (0.16 g, 10 times).

#### Hargreaves test

For testing heat sensitivity, we put mice in plastic boxes and measured the hindpaw withdrawal latency using Hargreaves radiate heat apparatus (IITCLife Science). The radiant heat intensity was adjusted so that basal paw withdrawal latency was between 9 and 15 s, with a cut-off of 20 s to prevent paw tissue damage.

### Immunohistochemistry

Spinal cord and DRG tissue section procedures were described in our previous report^19^. After blocking, the sections were incubated overnight at 4 °C with anti-MCP-1 (rabbit, 1:500; R&D Systems, Minneapolis, MN; mouse, 1:400; Thermo Fisher Scientific, Rockford, IL) or anti-IBA1 (rabbit, 1:2000; Wako, Osaka, Japan) primary antibodies, anti-NF-200 (mouse, 1:5000; Millipore, Billerica, MA), anti-CD68 (rat, 1:500; AbD Serotec, Raleigh, NC), and anti-F4/80 (rat, 1:500; eBioscience, San Diego, CA), followed by mixed FITC- and Cy3-conjugated secondary antibodies (1:400; Jackson ImmunoResearch, West Grove, PA). To improve MyD88 immune staining, antigen retrieval procedure was used. In brief, slides were first processed in citrate buffer (10 mM Aitric Acid, 0.05% Tween-20, pH6.0) at 95~100 °C for 30 min, and then the slides were incubated with anti-MyD88 antibody (rabbit, 1:1000; Abcam, Cambridge, MA) at 4 °C for overnight. For double immunofluorescence, sections were washed with PBS and then incubated with a mixture of polyclonal and monoclonal primary antibodies, followed by a mixture of Cy3- and FITC-conjugated secondary antibodies. For quantification of immunostaining, 3–6 DRG sections were selected from each animal and 4~6 animals were analyzed blindly in each group. To determine the percentage of labeled neurons in DRGs, the number of positive neurons (3 times of background staining) was divided by the total number of neurons. For IBA-1 staining, the number of positive cells was counted and the density of labeled cells (per square mm) was determined. Image-Pro Plus 5.0 software (Media Cybernetics, Silver Spring, MD) was used to analyze the images.

### Primary culture of DRG neurons and immunocytochemistry

Young mouse (4~6 weeks) DRGs were removed aseptically and incubated with collagenase (1.25 mg/ml)/dispase-II (2.4 units/ml) at 37 °C for 90 min, then digested with 0.25% trypsin at 37 °C for 8 min, followed by 0.25% trypsin inhibitor. Cells were mechanically dissociated with a flame polished Pasteur pipette in the presence of 0.05% DNAse I. DRG cells were placed on glass cover slips and grown in a neurobasal defined medium (with 2% B27 supplement) with 5 μM AraC and 5% carbon dioxide at 37 °C. DRG neurons were grown for 24 h before use. For immunocytochemistry, cultured DRG neurons were exposed to IL-1β (10 ng/ml in Opti-MEM) for 3 h, and then fixed with 4% paraformaldehyde for 20 min and followed by immunofluorescence process with anti-Myd88 antibody (rabbit, 1:200; Cell Signal, Beverly, MA). After immunostaining, 4′, 6′-diamidino-2-phenylindole (DAPI; 0.1 mg/ml) was added at room temperature for 5 min to stain all the nuclei of cells. The stained sections were examined with a Nikon fluorescence microscope, and images were captured with a CCD Spot camera. Five random regions from each cultured slice, total 4 slices per group were examined. The intensity of fluorescence was analyzed using ImageJ software (NIH; Bethesda, MD).

### Statistical analyses

All data were expressed as mean ± S.E.M. Data were analyzed using Student’s *t*-test (two groups), one-way or two-way ANOVA followed by post-hoc Bonferroni test with GraphPad Prism 5.0 software (La Jolla, CA) and were considered to be statistically significant at *P* < 0.05.

## Additional Information

**How to cite this article**: Liu, X.-J. *et al*. TLR signaling adaptor protein MyD88 in primary sensory neurons contributes to persistent inflammatory and neuropathic pain and neuroinflammation. *Sci. Rep.* **6**, 28188; doi: 10.1038/srep28188 (2016).
